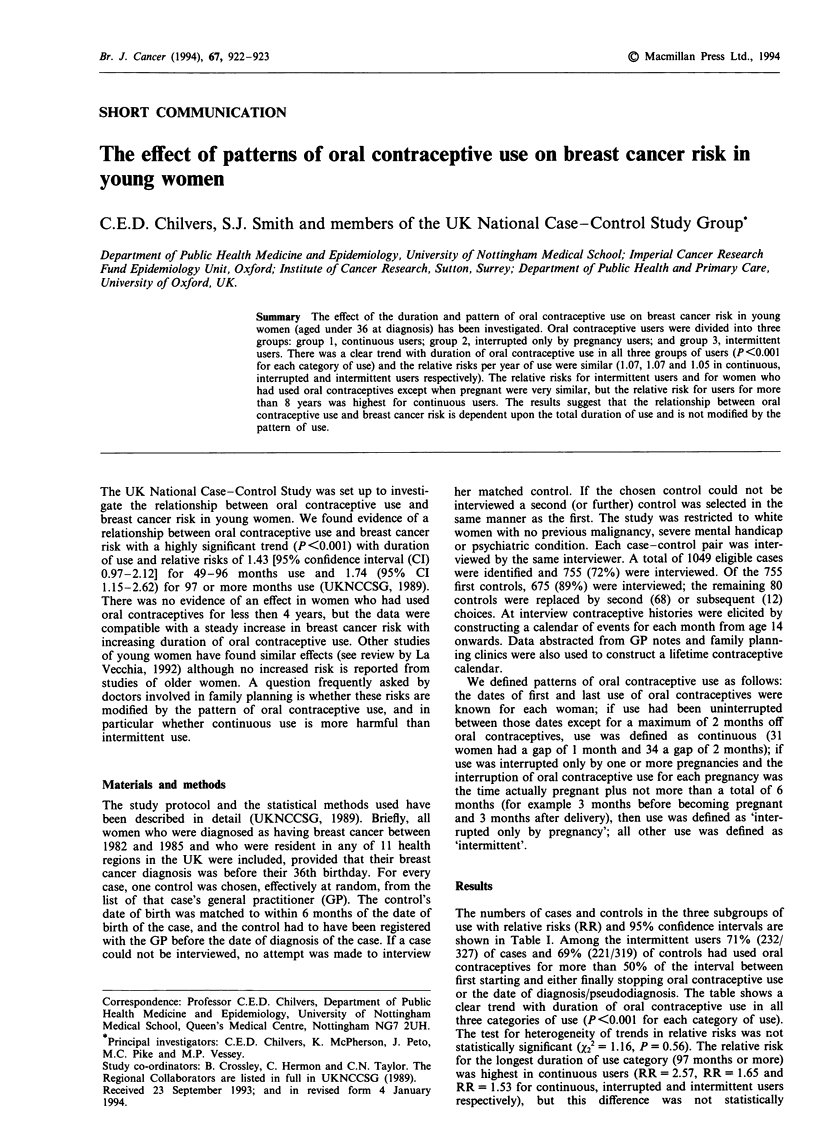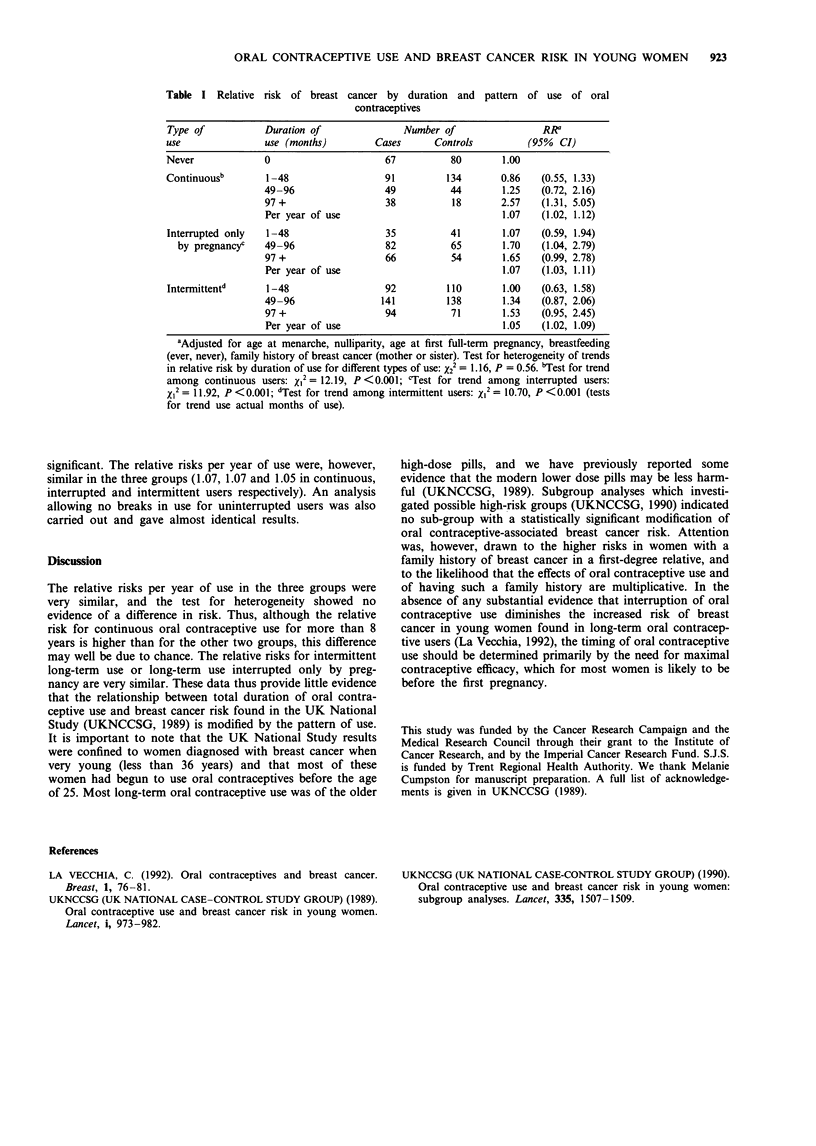# The effect of patterns of oral contraceptive use on breast cancer risk in young women. The UK National Case-Control Study Group.

**DOI:** 10.1038/bjc.1994.178

**Published:** 1994-05

**Authors:** C. E. Chilvers, S. J. Smith

**Affiliations:** Department of Public Health Medicine and Epidemiology, University of Nottingham Medical School, UK.

## Abstract

The effect of the duration and pattern of oral contraceptive use on breast cancer risk in young women (aged under 36 at diagnosis) has been investigated. Oral contraceptive users were divided into three groups: group 1, continuous users; group 2, interrupted only by pregnancy users; and group 3, intermittent users. There was a clear trend with duration of oral contraceptive use in all three groups of users (P < 0.001 for each category of use) and the relative risks per year of use were similar (1.07, 1.07 and 1.05 in continuous, interrupted and intermittent users respectively). The relative risks for intermittent users and for women who had used oral contraceptives except when pregnant were very similar, but the relative risk for users for more than 8 years was highest for continuous users. The results suggest that the relationship between oral contraceptive use and breast cancer risk is dependent upon the total duration of use and is not modified by the pattern of use.


Br. J. Cancer (1994), 67, 922-923 © Macmillan Press Ltd., 1994

SHORT COMMUNICATION

### The effect of patterns of oral contraceptive use on breast cancer risk in young women

C.E.D. Chilvers, S.J. Smith and members of the UK National Case-Control Study Group*

*Department of Public Health Medicine and Epidemiology, University of Nottingham Medical School; Imperial Cancer Research Fund Epidemiology Unit, Oxford; Institute of Cancer Research, Sutton, Surrey; Department of Public Health and Primary Care, University of Oxford, UK.*

**Summary** The effect of the duration and pattern of oral contraceptive use on breast cancer risk in young women (aged under 36 at diagnosis) has been investigated. Oral contraceptive users were divided into three groups: group 1, continuous users; group 2, interrupted only by pregnancy users; and group 3, intermittent users. There was a clear trend with duration of oral contraceptive use in all three groups of users ($P<0.001$ for each category of use) and the relative risks per year of use were similar (1.07, 1.07 and 1.05 in continuous, interrupted and intermittent users respectively). The relative risks for intermittent users and for women who had used oral contraceptives except when pregnant were very similar, but the relative risk for users for more than 8 years was highest for continuous users. The results suggest that the relationship between oral contraceptive use and breast cancer risk is dependent upon the total duration of use and is not modified by the pattern of use.

The UK National Case-Control Study was set up to investigate the relationship between oral contraceptive use and breast cancer risk in young women. We found evidence of a relationship between oral contraceptive use and breast cancer risk with a highly significant trend ($P<0.001$) with duration of use and relative risks of 1.43 [95% confidence interval (CI) 0.97-2.12] for 49-96 months use and 1.74 (95% CI 1.15-2.62) for 97 or more months use (UKNCCSG, 1989). There was no evidence of an effect in women who had used oral contraceptives for less then 4 years, but the data were compatible with a steady increase in breast cancer risk with increasing duration of oral contraceptive use. Other studies of young women have found similar effects (see review by La Vecchia, 1992) although no increased risk is reported from studies of older women. A question frequently asked by doctors involved in family planning is whether these risks are modified by the pattern of oral contraceptive use, and in particular whether continuous use is more harmful than intermittent use.

### Materials and methods

The study protocol and the statistical methods used have been described in detail (UKNCCSG, 1989). Briefly, all women who were diagnosed as having breast cancer between 1982 and 1985 and who were resident in any of 11 health regions in the UK were included, provided that their breast cancer diagnosis was before their 36th birthday. For every case, one control was chosen, effectively at random, from the list of that case's general practitioner (GP). The control's date of birth was matched to within 6 months of the date of birth of the case, and the control had to have been registered with the GP before the date of diagnosis of the case. If a case could not be interviewed, no attempt was made to interview her matched control. If the chosen control could not be interviewed a second (or further) control was selected in the same manner as the first. The study was restricted to white women with no previous malignancy, severe mental handicap or psychiatric condition. Each case-control pair was interviewed by the same interviewer. A total of 1049 eligible cases were identified and 755 (72%) were interviewed. Of the 755 first controls, 675 (89%) were interviewed; the remaining 80 controls were replaced by second (68) or subsequent (12) choices. At interview contraceptive histories were elicited by constructing a calendar of events for each month from age 14 onwards. Data abstracted from GP notes and family planning clinics were also used to construct a lifetime contraceptive calendar.

We defined patterns of oral contraceptive use as follows: the dates of first and last use of oral contraceptives were known for each woman; if use had been uninterrupted between those dates except for a maximum of 2 months off oral contraceptives, use was defined as continuous (31 women had a gap of 1 month and 34 a gap of 2 months); if use was interrupted only by one or more pregnancies and the interruption of oral contraceptive use for each pregnancy was the time actually pregnant plus not more than a total of 6 months (for example 3 months before becoming pregnant and 3 months after delivery), then use was defined as 'interrupted only by pregnancy'; all other use was defined as 'intermittent'.

### Results

The numbers of cases and controls in the three subgroups of use with relative risks (RR) and 95% confidence intervals are shown in Table I. Among the intermittent users 71% (232/327) of cases and 69% (221/319) of controls had used oral contraceptives for more than 50% of the interval between first starting and either finally stopping oral contraceptive use or the date of diagnosis/pseudodiagnosis. The table shows a clear trend with duration of oral contraceptive use in all three categories of use ($P<0.001$ for each category of use). The test for heterogeneity of trends in relative risks was not statistically significant ($\chi_2^2 = 1.16$, $P = 0.56$). The relative risk for the longest duration of use category (97 months or more) was highest in continuous users (RR = 2.57, RR = 1.65 and RR = 1.53 for continuous, interrupted and intermittent users respectively), but this difference was not statistically

Correspondence: Professor C.E.D. Chilvers, Department of Public Health Medicine and Epidemiology, University of Nottingham Medical School, Queen's Medical Centre, Nottingham NG7 2UH.

*Principal investigators: C.E.D. Chilvers, K. McPherson, J. Peto, M.C. Pike and M.P. Vessey.

Study co-ordinators: B. Crossley, C. Hermon and C.N. Taylor. The Regional Collaborators are listed in full in UKNCCSG (1989).

Received 23 September 1993; and in revised form 4 January 1994.

**Table I** Relative risk of breast cancer by duration and pattern of use of oral contraceptives

| *Type of use* | *Duration of use (months)* | *Number of Cases* | *Number of Controls* | *RR*[a] | *(95% CI)* |
|---|---|---|---|---|---|
| Never | 0 | 67 | 80 | 1.00 | |
| Continuous[b] | 1–48 | 91 | 134 | 0.86 | (0.55, 1.33) |
| | 49–96 | 49 | 44 | 1.25 | (0.72, 2.16) |
| | 97 + | 38 | 18 | 2.57 | (1.31, 5.05) |
| | Per year of use | | | 1.07 | (1.02, 1.12) |
| Interrupted only by pregnancy[c] | 1–48 | 35 | 41 | 1.07 | (0.59, 1.94) |
| | 49–96 | 82 | 65 | 1.70 | (1.04, 2.79) |
| | 97 + | 66 | 54 | 1.65 | (0.99, 2.78) |
| | Per year of use | | | 1.07 | (1.03, 1.11) |
| Intermittent[d] | 1–48 | 92 | 110 | 1.00 | (0.63, 1.58) |
| | 49–96 | 141 | 138 | 1.34 | (0.87, 2.06) |
| | 97 + | 94 | 71 | 1.53 | (0.95, 2.45) |
| | Per year of use | | | 1.05 | (1.02, 1.09) |

[a]Adjusted for age at menarche, nulliparity, age at first full-term pregnancy, breastfeeding (ever, never), family history of breast cancer (mother or sister). Test for heterogeneity of trends in relative risk by duration of use for different types of use: $\chi_2^2 = 1.16$, $P = 0.56$. [b]Test for trend among continuous users: $\chi_1^2 = 12.19$, $P < 0.001$; [c]Test for trend among interrupted users: $\chi_1^2 = 11.92$, $P < 0.001$; [d]Test for trend among intermittent users: $\chi_1^2 = 10.70$, $P < 0.001$ (tests for trend use actual months of use).

significant. The relative risks per year of use were, however, similar in the three groups (1.07, 1.07 and 1.05 in continuous, interrupted and intermittent users respectively). An analysis allowing no breaks in use for uninterrupted users was also carried out and gave almost identical results.

### Discussion

The relative risks per year of use in the three groups were very similar, and the test for heterogeneity showed no evidence of a difference in risk. Thus, although the relative risk for continuous oral contraceptive use for more than 8 years is higher than for the other two groups, this difference may well be due to chance. The relative risks for intermittent long-term use or long-term use interrupted only by pregnancy are very similar. These data thus provide little evidence that the relationship between total duration of oral contraceptive use and breast cancer risk found in the UK National Study (UKNCCSG, 1989) is modified by the pattern of use. It is important to note that the UK National Study results were confined to women diagnosed with breast cancer when very young (less than 36 years) and that most of these women had begun to use oral contraceptives before the age of 25. Most long-term oral contraceptive use was of the older high-dose pills, and we have previously reported some evidence that the modern lower dose pills may be less harmful (UKNCCSG, 1989). Subgroup analyses which investigated possible high-risk groups (UKNCCSG, 1990) indicated no sub-group with a statistically significant modification of oral contraceptive-associated breast cancer risk. Attention was, however, drawn to the higher risks in women with a family history of breast cancer in a first-degree relative, and to the likelihood that the effects of oral contraceptive use and of having such a family history are multiplicative. In the absence of any substantial evidence that interruption of oral contraceptive use diminishes the increased risk of breast cancer in young women found in long-term oral contraceptive users (La Vecchia, 1992), the timing of oral contraceptive use should be determined primarily by the need for maximal contraceptive efficacy, which for most women is likely to be before the first pregnancy.

This study was funded by the Cancer Research Campaign and the Medical Research Council through their grant to the Institute of Cancer Research, and by the Imperial Cancer Research Fund. S.J.S. is funded by Trent Regional Health Authority. We thank Melanie Cumpston for manuscript preparation. A full list of acknowledgements is given in UKNCCSG (1989).

### References

LA VECCHIA, C. (1992). Oral contraceptives and breast cancer. *Breast*, **1**, 76–81.

UKNCCSG (UK NATIONAL CASE-CONTROL STUDY GROUP) (1989). Oral contraceptive use and breast cancer risk in young women. *Lancet*, **i**, 973–982.

UKNCCSG (UK NATIONAL CASE-CONTROL STUDY GROUP) (1990). Oral contraceptive use and breast cancer risk in young women: subgroup analyses. *Lancet*, **335**, 1507–1509.